# Influences on Infrared Thermography of the Canine Eye in Relation to the Stress and Arousal of Racing Greyhounds

**DOI:** 10.3390/ani11010103

**Published:** 2021-01-06

**Authors:** Belle Elias, Melissa Starling, Bethany Wilson, Paul McGreevy

**Affiliations:** Faculty of Science, Sydney School of Veterinary Science, University of Sydney, Sydney, NSW 2006, Australia; melissa.starling@sydney.edu.au (M.S.); bethany.wilson@sydney.edu.au (B.W.); paul.mcgreevy@sydney.edu.au (P.M.)

**Keywords:** arousal, dog, eye temperature, greyhound, infrared thermography, lacrimal caruncle, stress

## Abstract

**Simple Summary:**

To improve the welfare of racing greyhounds, the identification of stressful industry practices is required. One potential method for monitoring stress in greyhounds is infrared thermography, which measures surface temperature. This article reports on the use of eye temperature, which may increase after a stressful event. The location on the eye that temperature is taken from is likely to be critical to the measurements. This study monitored 465 greyhounds racing at three racetracks in New South Wales. It found that the right eye and lacrimal caruncle (the inner corner of the eye) revealed temperature changes most effectively. Eye temperatures increased after racing, which may be due to physical exertion, stress and arousal. Additionally, eye temperature was higher in dogs that waited longer to race. Dogs at Richmond racetrack had lower eye temperatures before racing, but higher eye temperatures after racing compared to those at the Wentworth Park and Gosford racetrack. Other factors that increased eye temperature included humidity and a dog’s coat colour, age and final placing. Greyhounds that have light-coloured coats, are younger or placed poorly may be more stressed after racing. These factors need to be considered so that stress can be accurately detected.

**Abstract:**

Infrared thermography (IRT) can be used to identify stressors associated with greyhound racing procedures. However, factors unrelated to stress may influence measurements. Validation of an eye side (right or left) and a reference point on the eye is required if IRT is to be standardised for industry use. Infrared images of greyhound heads (*n* = 465) were taken pre-racing and post-racing at three racetracks. Average temperature was recorded at seven different locations on each eye. A multivariate analysis model determined how several factors influenced eye temperature (ET) pre-racing and post-racing. As expected, ET increased after racing, which may be attributed to physical exertion, stress and arousal. The right eye and lacrimal caruncle had the highest sensitivity to temperature changes and could be considered reference points for future studies. Additionally, dogs that raced later had higher ET, and Richmond racetrack had the lowest pre-race ET, but the highest post-race ET. This may suggest that arousal increases as the race meet progresses and certain track attributes could increase stress. Furthermore, ET increased as humidity increased, and higher post-race ET was associated with light-coloured, young and low-performing dogs. Environmental and biological factors need to be considered if IRT is to become accurate in the detection of canine stress and monitoring of greyhound welfare.

## 1. Introduction

There has been an increase in public concern regarding the welfare of racing greyhounds in Australia. This has threatened the ongoing public acceptance of the industry’s standard practices and its social license to operate. The science of greyhound welfare is an area of growing interest, with a focus on stress associated with the race and race meet protocols [1,2,3,4]. Stress can compromise an animal’s optimal state of homeostasis and wellbeing [5]. Shifts in the animal’s ability to cope may manifest as a behavioural, physiological or psychological response to an external or internal stimulus [5]. The stress response can become detrimental to health if it becomes chronic, which can occur via repetition and persistence of an aversive stimulus [6]. The effects of stressors on affective state vary in their valence (from pleasant to unpleasant) and with the animal’s current level of arousal (from deactivated to activated). There is evidence that greyhounds exhibit signs of physiological stress and arousal during a race meet [1,2,3], with arousal ranging from calm to alert, or excited [7,8]. Best practice in housing and managing greyhounds during a race meet should include optimising arousal and affective state at the time of maximal exertion, as well as the early detection and mitigation of stress.

Infrared thermography (IRT) has emerged as a non-invasive and reliable method to infer states of physiological stress in working [9,10], companion [11,12] and production animals [13,14]. It detects infrared radiation that is emitted from an object as a measure of its surface temperature (ST) and presents this as a heat map [11]. Recent studies have used IRT to measure the temperature of the eye, which may reflect how body temperature changes in response to events and stimuli [10,11,15,16,17,18]. Many IRT studies have shown that eye temperature (ET) has the highest correlation with core body temperature (CBT) when compared to the ST of other anatomical regions [10,11,16,17,18,19]. This correlation is attributed to the proximity of the orbit to the brain, as well as the rich blood supply it receives.

A decrease in ET may reflect a stress response that is dominated by the sympathetic nervous system (SNS) [20]. Studies of horses [9] and cattle [20] suggest that ET will decrease when the animals are startled or in pain, respectively. This decrease may represent an acute stress response whereby activation of the SNS mediates vasoconstriction and alters capillary flow [20]. Canine studies have highlighted that ET will decrease after dogs are separated from their owners, but will increase once they are reunited [12]. ET will also increase when undergoing a veterinary examination [11] or in anticipation of receiving a reward [19]. An increase of ET may reflect a stress response that is dominated by the parasympathetic nervous system (PNS) [21,22]. This may represent a less acute stress response whereby the hypothalamic–pituitary–adrenal (HPA) axis is activated and mediates peripheral vasodilation. An increase in ET may be consistent with the general stress of a race meet, including transportation, kennelling, race anticipation and the race itself.

Information about ET changes has been gained through the routine use of a particular reference point on the eye: the lacrimal caruncle [9,11,15,20,23,24]. However, the use of the lacrimal caruncle largely reflects convention. IRT studies justify its use as an unmistakable landmark that has the highest temperature on the eye [20]. However, the fact that this landmark has the highest temperature on the eye does not ensure that it will be the most effective location to either detect temperature changes or reflect CBT. There is also a need to establish whether one eye (right or left) is more sensitive to temperature changes after a stimulus. Establishing this would determine whether the temperature of one or both eyes must be recorded in future studies. An IRT study of stress responses of endurance horses found a positive correlation between the temperature of the right eye and the left eye after training [25]. However, this relationship has not been explored in dogs, so it will be assessed in the current study with the use of greyhound ET during a race meet.

Research indicates that dogs display both behavioural and physiological signs of stress during ground and air travel [26,27]. One study showed that plasma and salivary cortisol concentrations in dogs increased after ground transport [27]. Transport is one of many potential stressors that a greyhound may encounter during a race meet. In Australia, competing greyhounds generally travel in air-conditioned trailers. Additionally, they are kennelled in air-conditioned buildings at the race meet. Kennelled dogs may display signs of behavioural stress, including increased vocalisation, that correlate with signs of physiological stress, including elevated cortisol concentrations [28]. This may be due to the unpredictable nature of the kennelling environment and the potential social interactions that may occur [29].

Studies confirm that anticipation of a stimulus, such as a reward or race, may increase arousal levels in dogs [19,28,30]. One such stimulus for greyhounds is the so-called stir-up, which is a feature of Australian greyhound racing. The stir-up occurs approximately five minutes before each race and revolves around the greyhounds being allowed to watch others compete. One study reported that greyhounds had elevated arousal levels by merely watching others race, as well as when they raced themselves [1]. The use of a lure and catching pen in races may further contribute to the greyhounds’ arousal and, to some extent, their frustration. During a race, the greyhounds will chase the lure as it proceeds along the track. Catching pens are then used to divert the greyhounds away from the lure and into a holding area at the end of the race [11]. Catching pens are only used within Australia and are intended to decrease the risk of injury [31]. However, the thwarted motivation to prehend the putative prey item (the lure) can result in stress-related frustration, as the greyhounds are unable to capture or interact with it [31]. So-called teasers, with which dogs are allowed to interact, are now being trialled at some tracks in Australia in a bid to avoid stress-related frustration. A teaser is a toy attached to a bungee line, which will spring back into the catching pen as the dogs approach the end of the race.

Along with stressors associated with the race meet, there are other environmental and biological factors that could influence the IRT measurements and must be considered when assessing stress and arousal [32,33]. Ambient temperature and humidity could be sources of stress during a race, as these factors can compromise an animal’s ability to thermoregulate [2,34,35]. A previous greyhound study found that rectal temperature (RT) increased during high ambient temperatures [2]. It suggested that the risk of heat stress was higher among greyhounds with dark-coloured coats [2]. Coat colour is important for heat resistance, given that research on cattle [36] and sheep [37] suggests light-coloured animals can maintain lower body temperatures than dark-coloured animals during heat stress. Dark-coloured coats may absorb more thermal radiation than light-coloured coats, and thus may increase ST and CBT [2,37,38].

In IRT studies, age has been reported to influence ST in cattle, with younger animals retaining lower ST than older animals [39]. Human studies suggest that ET decreases with age [40]. However, relationships between ET and age have not been reported in dogs. In cattle, sex can alter body temperature, especially when a cow is in oestrus [41]. In New South Wales (NSW), a greyhound bitch in season is not allowed on the premises during a race meet. This provision may reduce the effects of sex on body temperature during a race. However, one study found that male greyhounds had a higher RT after racing, when compared to females [2]. It also reported a positive interaction between body weight and increased RT after racing. Heat is generated as a by-product of ATP production and utilisation during muscular activity [2], so the male effect may reflect their larger bodies. The larger muscle mass typical of males is expected to produce more metabolic heat [2]. Furthermore, an animal’s fitness and racing performance may influence IRT measurements, given that an equine study showed that high-placing racehorses had higher ST than low-placing racehorses [42].

The current study presents further analysis of data first reported in the preliminary paper by Starling et al. (2020) [43]. It was designed to identify which eye side (right or left) and reference point on the eye had the highest sensitivity to temperature changes after exposure to a stimulus. It also investigates race meet factors that may be associated with a physiological stress response, as shown through an increase of ET. Furthermore, the environmental and biological factors that influence IRT measurements of the eye are assessed, because these factors may confound the detection of stress associated with the race and race meet protocols.

## 2. Materials and Methods

Data for this study were collected under the approval of the University of Sydney Animal Ethics Committee (approval number: 2016/1015).

### 2.1. Location

The data were collected in 2017 from 3 racetracks in NSW. In June, data were collected at the Richmond track during 3 race meets, with 11 races occurring per meet. In July, data were collected at the Wentworth Park track during 2 race meets, with 10 races occurring per meet. In October and November, data were collected at the Gosford track during 3 race meets, with 8, 10 and 11 races being recorded, respectively. The ambient temperature and relative humidity at these locations were recorded to the nearest hour of each race, using the records from Time and Date AS [44]. This website obtains relevant meteorological information from Custom Weather [45].

During all of the recorded races at Richmond, a bungee teaser was being trialled. The Wentworth Park track and Gosford track did not use teasers during the races. The teaser at Richmond was comprised of two toys attached to separate bungee lines. Each toy was covered in synthetic fur. The teaser was released as the competing dogs approached the catching pen at the end of the race. It moved along the track and the dogs could follow it into the sand trap within the catching pen, where they could interact with it.

### 2.2. Subjects

A total of 465 greyhounds were studied: 185 at Richmond, 127 at Wentworth Park and 153 dogs at Gosford. Data were not collected on dogs that had already been studied during a previous race or race meet. The dogs were given a 30 s physical examination by a veterinarian once they arrived at the race meet. All dogs deemed fit to race were then kennelled in an air-conditioned building awaiting the pre-stir-up. Each dog was given enough space in its kennel to stand up and lie down. During the race meet, greyhound handlers were asked a series of questions about their dog and their dog’s racing history. These questions included:

1. How old is your greyhound (rounding to the nearest half year)?
2. How many times has your greyhound raced?
3. How many days has it been since your greyhound last raced?
4. How many minutes did it take to travel to the racetrack with your greyhound?

All of the information gathered during the race meets was cross-checked against, and collated with, data from “The Dogs” website, which is the official website for greyhound racing in Australia [46]. Data pertaining to the race (date, time, distance and number) were recorded, as well as data about each greyhound (starting box number, sex, age, weight, coat colour and final placing). Coat colours were divided into a “light” or “dark” category using the methodology from a previous greyhound study [2]: light-coloured dogs had coats that were fawn or white (including white mixes that were predominantly white); dark-coloured dogs had coats that were predominantly black, blue or brindle.

### 2.3. Data Collection

As per the ethics approval, all greyhound handlers consented to the collection of infrared images of the competing dogs. When permitted by the handlers, the dogs had two infrared images taken; one 10 min before the race during the pre-stir-up and another 15 min after the race had finished. Before the race, the dogs were removed from their kennels for the pre-stir-up and were given the opportunity to eliminate next to the track. The first image was taken during this time, as it was the closest period to the race when all dogs were accessible. After the race, the dogs were hosed down and offered water before being kennelled again. They were required to stay in the kennels for at least 15 min, after which they could be removed to vehicles and taken home. The second infrared image was taken at the end of this 15-min post-race kennelling period. This reduced the effects of physical exertion on ET, while still ensuring post-race images could be collected for as many dogs as possible.

A FLIR T640 Professional Thermal Imaging Camera (T640, FLIR Systems Inc., Danderyd, Sweden) was used to capture the images. This device was calibrated before the study period began. The thermal images were taken out of direct sunlight and wind. They were taken 1 metre away from each greyhound at an 80–100⁰ angle from the midline of the dog’s head. Opportunistically, the images focused on either the right or left eye, or both if possible (Table 1). There were 111 dogs with images of the right eye captured pre- and post-racing, 123 dogs with images of the left eye captured pre- and post-racing, and 21 dogs with images of both the right and left eye captured pre- and post-racing.

**Figure 1:**
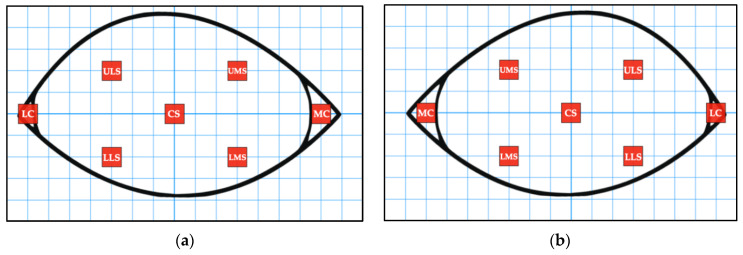
(**a**) The seven locations where the Region of Interest averages were recorded on the right eye. (**b**) The seven locations where the Region of Interest averages were recorded on the left eye.

**Figure 2:**
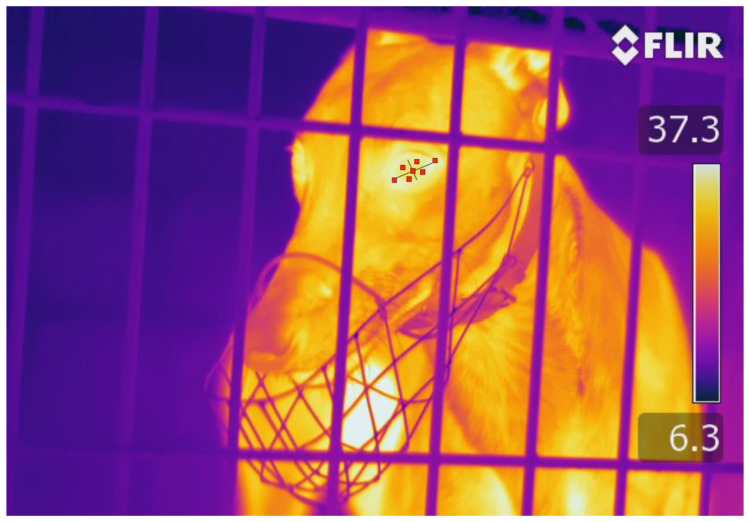
Infrared image of a greyhound viewed using the ResearchIR Max software. The red boxes on the left eye represent the seven locations in which the Region of Interest averages were recorded. The centre of the eye was located at the intercept of the black lines.

The infrared images were analysed using the FLIR ResearchIR Max software (FLIR Systems Inc., Danderyd, Sweden). The 1234 palette was used for each image as it provided good contrast, allowing the eye to be viewed clearly. The Measurement Cursor Region of Interest (ROI) was used to read the average temperature of a 3 × 3 box of pixels. This ROI was chosen to provide a more accurate indication of temperature as it reads the average of 9 pixels, rather than the Spot Cursor ROI which reads the value of a single pixel. Measurements were undertaken for seven different locations on each eye, including the medial canthus (specifically the lacrimal caruncle) (MC), lateral canthus (LC), upper surface of the eye closest to the MC (UMS), upper surface of the eye closest to the LC (ULS), lower surface of the eye closest to the MC (LMS), lower surface of the eye closest to the LC (LLS) and the centre surface of the eye (CS) (Figure 1 and Figure 2). Where possible, all seven ROI averages were recorded. However, some ROI averages were missing due to obstructions, such as kennel bars, or the angle of the dog’s head within the image (Table 2). If multiple ROI averages were not apparent on a given image, the image could be discarded. Furthermore, blurry images were not used as accurate temperature measurements would not be obtained.

### 2.4. Statistical Analysis

The mean distance travelled during races, mean ambient temperature and mean relative humidity across the dataset were calculated using Microsoft Excel for Mac (Version 16.40, Microsoft, Redmond, WA, USA). The range, mean and standard deviation of ambient temperature and relative humidity at each racetrack were also calculated.

The statistical analysis was performed in RStudio Cloud (version 1.2.5033.1, Mac OS, RStudio Inc., Boston, MA, USA). A univariate analysis was performed for each variable. Variables with a *p*-value higher than 0.2 were excluded from the multivariate analysis. Excluded variables included the distance travelled to the race meet (Road_Travel), the time of day that the race occurred (Race_Time) and the box number that the dog started the race from (Starting_Box). Age and sex had *p*-values higher than 0.2, but were forced into the multivariate analysis based on the current literature [2,39,40] and to facilitate the interpretation of results.

A mixed model approach was chosen to permit inclusion of a dog effect and to model the repeatability of the data. The final additive multivariate analysis model was built using the stepwise deletion method and a significance level of 0.05. Linear mixed models were fit by maximum likelihood, and t-tests were calculated using the Satterthwaite’s method. Potential interactions were explored based on the final additive model and the Akaike Information Criterion (AIC) was used to determine the model of best fit. The factors excluded from the final model by the stepwise deletion method included how many times the dog had previously raced (Racing_Starts), the number of days since the dog last raced (Days_Last_Raced), distance travelled during the race (Race_Distance) and ambient temperature (Ambient_Temp). Ambient temperature was not forced into the model due to the effects of multicollinearity when including data from the two environmental factors: ambient temperature and relative humidity (Humidity). This would produce an overly complicated model that must be avoided. Relative humidity was included in the final additive model, as this environmental factor produced a more effective model based on the results of the stepwise deletion method.

## 3. Results

### 3.1. Descriptive Statistics

A total of 277 dogs had images taken both before and after the race, whereas 161 dogs had images taken only before the race and 27 dogs had images taken only after the race. Throughout the study period, all recorded races occurred between 3:12 p.m. and 10:42 p.m. The study population included male and female greyhounds between the ages of 1 to 6 years. The weight of these dogs ranged between 21.9 kg and 39.1 kg. In this study, 17% of dogs (*n* = 77) had light-coloured coats and 83% of dogs (*n* = 388) had dark-coloured coats. The racing experience varied among individuals, with the number of racing starts ranging from 0 to 177 and the days since last raced ranging from 1 to 200. The dogs travelled between 1 to 430 min by road to the racetrack. The distance travelled during races at Gosford track ranged from 400 metres to 600 metres, averaging at 473.77 metres. Richmond track had races that ranged from 330 metres to 618 metres, averaging at 444.5 metres. Wentworth Park track had races that ranged from 520 metres to 720 metres, averaging at 537.65 metres. Furthermore, ambient temperature ranged from 10 °C to 18 °C, averaging at 13.9 °C, while relative humidity ranged from 42% to 87%, averaging at 66.4%. The environmental conditions were not confounded between racetrack and season, as shown in Table 3.

### 3.2. Model Factors

A total of 5696 ET observations from 457 dogs were included in the final model. The additive factors in the final model (AIC: 16743.8, Bayesian Information Criterion (BIC): 16876.8) included whether the image was taken before or after the race (Image_Timing), whether the temperature was taken from the right or left eye (Eye_Side), the location on the eye in which the temperature was taken from (Eye_Location), the order of the race during each race meet (Race), the track the race was held at (Track) and the relative humidity. The final model also included the dog’s coat colour (Coat_Colour), age (Age), sex (Sex), weight (Weight) and final placing (Performance). It was hypothesised that these additive factors would have a significant effect on ET. Furthermore, the final model had seven interactions: Image_Timing and Eye_Side, Image_Timing and Eye_Location, Image_Timing and Track, Image_Timing and Humidity, Image_Timing and Coat_Colour, Image_Timing and Age, and Image_Timing and Performance. These interactions were added to explain more variance and decrease the AIC (AIC: 16468.4, BIC: 16687.8).

### 3.3. Imaging and Measurement Factors

There was a significant difference between ET before and after the race (*F*-value: 14.001, *p*-value: <0.001). This corresponded to an increase of ET after the race for the reference class (dark-coloured dogs, at the MC) (reference effect: 1.041, standard error (SE): 0.203, *t*-value: 5.120, *p*-value: <0.001).

**Figure 3:**
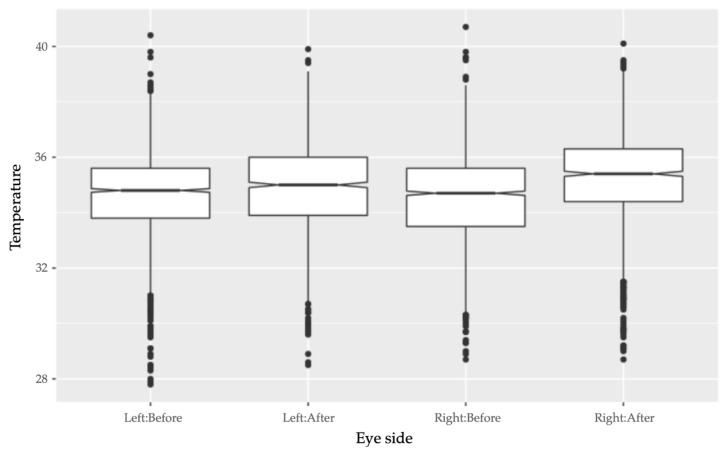
Boxplot showing how temperatures of both the right and left eyes increased after racing. Compared to the left eye, the right eye had a higher increase in temperature after the race.

Eye side did not have a significant effect on pre-race ET (*F*-value: 1.273, *p*-value: 0.259). However, eye side did have a significant positive interaction with image timing, which corresponded to an increase of both right and left ET after the race. There was a significantly greater increase in ET for the right eye than for the left eye after the race (interaction effect: 0.151, SE: 0.062, *t*-value: 2.457, *p*-value: 0.014) (Figure 3).

**Figure 4:**
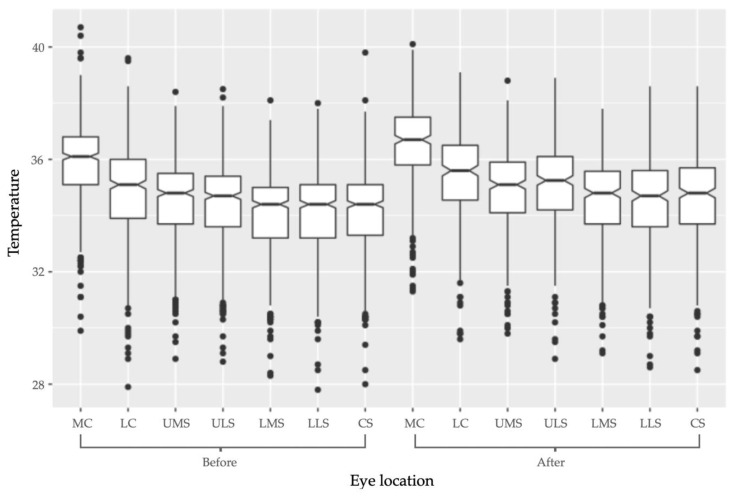
Boxplot of temperature change at the seven locations across the eye after racing. Both before and after racing the medial canthus had the highest temperature, followed by the lateral canthus.

Location of measurement had a significant effect on ET (*F*-value: 404.888, *p*-value: <0.001). The LC, UMS, ULS, LMS, LLS and the CS had significantly lower temperatures than the MC (Table 4). There was also an interaction between location and image timing (*F*-value: 2.709, *p*-value: 0.013). The MC had the highest temperature increase after racing whereas, for several other locations on the eye, this effect was significantly smaller (Figure 4). However, the temperature effects of the race at the LC and ULS were not significantly different from those at the MC (Table 5).

### 3.4. Race Meet Factors

Race number had a significant effect on ET. As the race number increased, ET increased (reference effect: 0.063, SE: 0.018, *t*-value: 3.436, *p*-value: <0.001). The track also had a significant effect on ET. Compared to Richmond, Gosford (reference effect: 1.214, SE: 0.146, *t*-value: 8.339, *p*-value: <0.001) and Wentworth Park (reference effect: 1.499, SE: 0.158, *t*-value: 9.466, *p*-value: <0.001) had higher pre-racing ET. There was no significant difference in pre-race ET at Wentworth Park compared to Gosford (reference effect: 0.285, SE: 0.212, *t*-value: 1.344, *p*-value: 0.180). Furthermore, the effect of the track on ET was greater after racing than before. Compared to Gosford, there was a higher increase of ET after racing at Richmond (interaction effect: 0.573, SE: 0.080, *t*-value: 7.204, *p*-value: <0.001). Similarly, compared to Wentworth Park, Richmond had a higher increase of ET after racing (interaction effect: 0.649, SE: 0.084, *t*-value: 7.679, *p*-value: <0.001). There was no significant difference in post-race ET at Wentworth Park compared to Gosford (interaction effect: –0.075, SE: 0.114, *t*-value: –0.660, *p*-value: 0.509).

### 3.5. Environmental Factors

Relative humidity had a significant effect on ET. For pre-race measurements, as humidity increased, ET also increased (reference effect: 0.011, SE: 0.005, *t*-value: 2.226, *p*-value: 0.026). The effect of humidity on ET was greater after racing than before, leading to a greater increase of ET due to humidity after the race (interaction effect: 0.013, SE: 0.003, *t*-value: 5.260, *p*-value: <0.001).

### 3.6. Biological Factors

Coat colour did not have a significant effect on ET before racing (*F*-value: 3.554, *p*-value: 0.060). However, there was a significant interaction between coat colour and image timing (*F*-value: 19.499, *p*-value: <0.001). There was a greater increase of ET in light-coloured dogs after racing than in dark-coloured dogs (interaction effect: 0.356, SE: 0.081, *t*-value: 4.416, *p*-value: <0.001).

Age did not have a significant effect on ET before racing (*F*-value: 2.107, *p*-value: 0.147). However, as with coat colour, there was a significant interaction between age and image timing. As the age increased, the ET increased less after racing (interaction effect: −0.138, SE: 0.039, *t*-value: −3.571, *p*-value: <0.001). Sex (*F*-value: 0.008, *p*-value: 0.929) and weight (*F*-value: 0.065, *p*-value: 0.798) did not have a significant effect on ET.

Performance did not have a significant effect on ET before racing (reference effect: 0.004, SE: 0.024, *t*-value: 0.168, *p*-value: 0.867). However, there was an interaction between performance and image timing. As the performance of the dog improved, ET after racing increased less (interaction effect: −0.131, SE: 0.014, *t*-value: −9.657, *p*-value: <0.001).

## 4. Discussion

This study found that ET was higher after the race than before. The right eye showed more flux in temperature changes than the left eye after racing. It was also found that the MC (specifically the lacrimal caruncle) had the highest sensitivity to temperature changes after racing. However, the LC and the ULS were not significantly different from the lacrimal caruncle. In terms of race meet factors, ET increased as the race number increased. Additionally, Richmond had the lowest ET pre-racing, but the highest ET increase post-racing. In terms of environmental factors, ET increased both pre- and post-racing as humidity increased. Furthermore, the biological factors that influenced post-race ET included coat colour, age and performance. Older, high-performing and dark-coloured dogs were less affected by the act of racing compared to younger, low-performing and light-coloured dogs, respectively.

### 4.1. Imaging and Measurement Factors

Dogs had higher ET after the race than before. This may be associated with physical exertion and exercise hypothermia, as IRT studies have shown that ET will increase as dogs exercise and exert energy [47,48]. This is due to the generation of heat from muscular activity. Exercise will also increase cardiac output and blood flow to the extremities [49]. There may also be an emotional response to racing that could be detected in ET, but this study was unable to distinguish between these potential influences. Future studies should consider post-race ET alongside CBT and behavioural indicators of stress.

There was no significant difference between the temperature of the right eye and the left eye before racing. However, the temperature of both eyes increased after racing, and there was a higher increase of post-race ET on the right eye than the left eye. This may suggest that the right eye was more sensitive to the effects of acute physical exertion or physiological arousal. This may reflect a lateral response to the single direction of racing, as the greyhounds were raced anticlockwise at each racetrack. Even so, the use of the right eye in future ET studies on canine stress may be preferred for revealing more change in temperature, especially if the changes to be detected are only marginal.

Furthermore, the location from which temperature measurements were taken on the eye had a significant influence on ET, both before and after racing. Each location was significantly different from the lacrimal caruncle before racing. The lacrimal caruncle had the highest temperature across the eye and the LC had the second highest temperature; a finding that supports the current literature [11]. This may be due to the rich capillary beds that surround the posterior border of the eyelid and the lacrimal caruncle [11,20]. Research has shown that the lacrimal caruncle responds to changes in blood flow and has been sensitive to CBT changes in response to SNS activation and HPA activation [11,20,21,22]. To these authors’ knowledge, no canine research has compared temperature sensitivity at different locations on the eye, including the lacrimal caruncle, across a sampling population.

This study is the first to establish that the lacrimal caruncle is the ocular location with the highest sensitivity to temperature changes after racing. This provides evidence to support the use of the lacrimal caruncle in IRT studies, as this location may be able to detect slight temperature changes more effectively than other ocular locations. Other locations that were measured on the eye included the LC, UMS, ULS, LMS, LLS and CS. The temperatures recorded at the LC and the ULS were not significantly different from those at the lacrimal caruncle. Therefore, these locations may be useful as proxy locations for ET measurements if the lacrimal caruncle is not apparent in a given opportunistic infrared image. That said, the lacrimal caruncle should be the preferred location at which ET measurements are taken in elective imaging studies. This is based on the finding that the other ocular locations had lower temperatures than the lacrimal caruncle after racing, and because the literature has shown that temperatures at this reference point are reflective of responses to both pain and other stressors [11,20,21,22].

### 4.2. Race Meet Factors

The race number had a positive association with pre-race ET; the longer the dogs had to wait to race, the higher their ET became. This may be due to the greyhounds becoming more aroused as the race meet progressed [43]. Heightened arousal may be attributed to ongoing disturbance during kennelling and anticipation of the race [19,28,30,43]. For further discussion, readers are directed to Starling et al. (2020) [43].

Pre-race ET was lower at the Richmond track, than at the Gosford track and Wentworth Park track. The preliminary paper suggests that the Gosford and Wentworth Park tracks may be inherently more stressful pre-racing than the Richmond track [43]. The environmental conditions were not confounded between racetrack and season. Therefore, the difference in pre-race ET could be due to kennel design or the handling techniques used to remove the dogs from the kennels for the pre-stir-up [43]. Again, readers are directed to Starling et al. (2020) [43] for further discussion.

In the current study, the effect of track was more important after the race. The differences in post-race ET between tracks are not easily explained. Results suggest environmental conditions were not the primary influence on post-race ET, as post-race ET at Gosford (October/November) and Wentworth Park (July) was not significantly different. Richmond (June) had the highest post-race ET, but the lowest mean ambient temperature (by <1 °C) and a mean humidity between that of Gosford and Wentworth Park. If environmental conditions were the primary influence on post-race ET, it would be expected that there would be a significant difference between Gosford and both Wentworth Park and Richmond, but a much smaller difference between Richmond and Wentworth Park. That this pattern was not found suggests the differences in post-race ET may be attributed to other track effects. Post-race ET was highest at the Richmond track, which could suggest that racing is more physically taxing at the Richmond track. This effect could also reflect the use of a teaser (a putative prey item) at this location alone, as studies have shown that a dog’s ET will increase in anticipation of receiving a food treat [19]. However, this is beyond the scope of the study and the use of a teaser at one location alone may confound the results.

### 4.3. Environmental Factors

Humidity had a significant effect on ET, which was greater after racing than before. This corresponded to a higher increase of post-race ET associated with humidity. This finding supports the current literature, which suggests that humidity may compromise canine thermoregulation, particularly during strenuous exercise [2,34,35]. Thermoregulation in dogs relies on evaporative cooling, primarily via panting [34,35]. Dogs generate heat during exercise, so their respiratory rates increase to drive heat transfer, chiefly from the mouth and upper airways [1,2]. Ideally, dogs at risk of hyperthermia will inhale dry and cool air, which optimises evaporative heat loss from the tongue, nasal mucosa and oral mucosa through the exhalation of moister and warmer air [34]. However, high levels of humidity, as in the current study, may restrict the amount of heat that can be lost during evaporative cooling [2,34,35]. Therefore, especially after exertion, dogs may become susceptible to heat stress and CBT may increase, which could account for the current increase in post-race ET. Ambient temperature may also affect evaporative cooling when it approaches or exceeds CBT [2,34,35]. It may cause heat stress by decreasing the heat gradient between the environment and the dog, as well as adding radiant heat to the animals [34]. Individual characteristics may also be of importance here, particularly the effects of coat colour on thermal absorption [2]. That said, ambient temperature was excluded from the final additive model using the stepwise deletion method.

### 4.4. Biological Factors

Coat colour did not have a significant effect on pre-race ET. However, a higher post-race ET was found in light-coloured dogs. In contrast to the literature [2], this may suggest that dark-coloured dogs were less affected by the act of racing than light-coloured dogs. A previous study of greyhounds (*n* = 229) reported that those with dark-coloured coats had higher post-exercise RT than those with light-coloured coats [2]. That study also found a positive association between ambient temperature and post-exercise RT, and suggested that the higher RT of dark-coloured dogs may be attributed to increased absorption of surrounding thermal radiation [2]. The ambient temperature during their study ranged from 11 °C to 40.8 °C [2], whereas, in the current study, it ranged from 10 °C to 18 °C. The lower temperature extreme in the current study may account for this apparent discrepancy as well as the exclusion of ambient temperature from the final additive model. The lower post-race ET for dark-coloured dogs may be associated with acclimatisation, in that the dark-coloured dogs are better adapted to additional heat stress, as they may be accustomed to heat stress training and racing during hotter days outside the study period. Therefore, they may be less susceptible than light-coloured dogs to changes in CBT, and therefore ET during racing in cooler temperatures. However, this is only one potential explanation and it is speculative.

Similar to coat colour, the age, sex, weight and performance of dogs did not have a significant effect on pre-race ET. However, there was an association between post-race ET and age. Greyhounds in this study ranged from 1 to 6 years of age. The increase of post-race ET was less pronounced in older dogs than in younger dogs. This may suggest that older dogs are less affected by racing than younger dogs. Young and inexperienced racehorses can quickly develop hyperthermia when they become overly aroused [50]. Furthermore, studies suggest that excitability and arousal levels may be higher in smaller and younger dogs [51,52,53,54]. Therefore, in the current study, the higher post-race ET in younger dogs may be associated with less heat resistance and/or higher arousal levels than in older, more seasoned dogs.

There was also an association between post-race ET and performance. The increase in post-race ET was less pronounced in dogs that placed higher in their races. This may suggest that high-placing dogs were less affected by racing than low-placing dogs. This prospect has not been reported in the literature. However, a racehorse study found that high-placing animals had higher ST than low-placing animals [42]. In the current study, high-placing dogs may be fitter and less susceptible to metabolic heat stress. However, when reporting on much fewer greyhounds (*n* = 229), McNicholl et al. (2016) [2] found no association between fitness level and post-race RT. The results of the current study may also suggest that older dogs and high performing dogs may cool more quickly, thus reducing the increase of post-race ET.

### 4.5. Study Limitations and Future Research

The first infrared image was taken before the race when the dogs were removed from kennelling for the pre-stir-up. Future studies should aim to take an additional infrared image prior to first kennelling, so that an approximate baseline temperature for each greyhound can be established. Although, this may not reflect a true baseline either, as greyhounds travel to the race meet and are quickly moved to a queue for vetting once they are unloaded from vehicles. However, an additional image may reveal more information about the effects of kennelling and the pre-stir-up. Additionally, the links between stress and race meet practices and protocols in the current study are largely speculative. They are presented here chiefly to generate avenues for further enquiry. Future studies should focus sampling on fewer dogs to allow for CBT measurements to be collected, as well as data from the same dogs at multiple tracks. Furthermore, IRT fails to discriminate between emotional valence and arousal [7,11,19]. Emotional valence describes the affective state, which ranges from negative to positive [7,8]. Greyhounds may exhibit behavioural signs of emotional stress during a race meet. Therefore, future studies should also use behavioural indicators, such as data scored against ethograms [11,19]. This would allow emotional stress to be distinguished from physiological arousal.

The use of CBT alongside ET would be beneficial in future studies to clearly identify non-exertional effects on temperature differences and identify the latency for ET to return to baseline temperatures after the race. A previous greyhound study reported that heart rate returned to pre-race values 10 min after a 100-metre sprint. However, that study also suggested that a longer recovery period may be required for RT to return to the pre-race temperature [3]. Another study found that after dogs have exercised for a 30-min period, CBT can take up to 30 min to return to baseline [47]. In the current study, the second infrared image was taken after a 15-min cool-down period. Therefore, physical exertion and exercise hyperthermia are likely to have played a significant role in post-race ET measurements and it would be difficult to isolate the effects of physiological stress. However, it was not possible to take the image more than 15 min after the race because, at that juncture, the greyhounds were being removed from the venues. There is a strict schedule that must be adhered to and handlers were concerned about the stress levels of their dogs. Future studies could sample dogs at training and trial events. However, this would not account for the effects of the race day environment.

In terms of eye side sensitivity, the current opportunistic study should be considered as a pilot study. The greyhounds were raced only in an anticlockwise direction at the three racetracks, which may mean there was a lateral influence. Therefore, future studies should investigate the sensitivity of the right and left eye of greyhounds racing in both directions. Furthermore, the association between coat colour and post-race ET during high ambient temperatures warrants further study, as the current study was conducted on relatively cool days, averaging at 13.9 °C. Future studies could also aim to obtain a more balanced ratio of light-coloured dogs and dark-coloured dogs, as 17% of dogs in this study were considered “light” and 83% of dogs were “dark”. A balanced population may permit a more detailed analysis of any differences between the two phenotypes.

## 5. Conclusions

IRT is a useful method to infer states of stress and arousal in canine welfare science. IRT can be used to measure the temperature of the eye to reflect how CBT changes in response to stressors. The use of the right eye and lacrimal caruncle should be implemented in future IRT studies, as they are particularly sensitive to temperature changes. Stressors related to racing may include the length of periods dogs spend waiting to race, as well as track attributes that warrant further study with CBT comparisons. Environmental and biological factors that may be unrelated to stress can influence IRT measurements of the eye due to the effects of physical exertion. These factors include humidity and a dog’s coat colour, age and final placing in the race. These factors need to be addressed and built into emerging welfare assessment protocols, so that stress can be accurately measured in future studies. Industry procedures and greyhound welfare could then be improved through the early detection and mitigation of stress. This may help to address the concerns of the public, as well as maintain racing integrity through the routine use of IRT to screen greyhounds for their fitness to race.

## Figures and Tables

**Table 1:** The distribution of infrared images that captured the right eye, left eye or both eyes, before and after the race.

Period	Eye Side Captured in Infrared Image	Number of Infrared Images
Before the race	Right eye only	148
	Left eye only	165
	Both right and left eye	124
After the race	Right eye only	99
	Left eye only	120
	Both right and left eye	81

**Table 2:** The number of Region of Interest averages recorded for each location on the eye, both before and after the race.

Period	Location on the Eye	Number of Recorded Region of Interest Averages
Before the race	MC	508
	LC	473
	UMS	516
	ULS	522
	LMS	506
	LLS	515
	CS	544
After the race	MC	304
	LC	269
	UMS	320
	ULS	313
	LMS	316
	LLS	301
	CS	337

**Table 3:** The minimum value, maximum value, mean and standard deviation of ambient temperature and relative humidity at each racetrack.

Environmental Factor	Racetrack	Month	Minimum Value	Maximum Value	Mean	Standard Deviation
Ambient temperature (°C)	Gosford	October/November	15	18	16.46	1.05
	Richmond	June	10	14	12.29	1.19
	Wentworth Park	July	11	15	13.17	1.41
Relative humidity (%)	Gosford	October/November	72	87	80.46	5.76
	Richmond	June	47	85	66.58	15.86
	Wentworth Park	July	42	59	49.09	5.85

**Table 4:** The distribution of estimates of reference effect, standard error, *t*-value and *p*-value of the different anatomical locations across the eye compared to the medial canthus.

Eye Location	Reference Effect	Standard Error	*t*-Value	*p*-Value
LC	−0.992	0.059	−16.850	<0.001
UMS	−1.316	0.058	−22.867	<0.001
ULS	−1.390	0.057	−24.218	<0.001
LMS	−1.810	0.058	−31.298	<0.001
LLS	−1.718	0.058	−29.826	<0.001
CS	−1.681	0.057	−29.611	<0.001

**Table 5:** Estimates of interaction effect, standard error, *t*-value and *p*-value of post-racing temperature at different anatomical locations across the eye compared to the medial canthus.

Eye Location	Interaction Effect	Standard Error	*t*-Value	*p*-Value
LC	−0.086	0.097	−0.880	0.379
UMS	−0.306	0.093	−3.272	0.001
ULS	−0.088	0.094	−0.934	0.350
LMS	−0.206	0.094	−2.192	0.028
LLS	−0.247	0.095	−2.613	0.009
CS	−0.229	0.092	−2.479	0.013

## Data Availability

The data presented in this study are available on request from the corresponding author.
